# Nasopharyngeal carcinoma detected noninvasively in the real world using three gene methylation analyses from automatically processed bilateral nasal swab samples

**DOI:** 10.1186/s12885-025-14508-y

**Published:** 2025-07-05

**Authors:** Zi-Han Qin, Si-Yuan Chen, Shuai Zhou, Hua Deng, Lan-Xi Li, Qi-Lun Guo, Xiong Zou, Pei-Yu Huang, Ming-Yuan Chen, Liang Zhang, Yi-Jun Hua

**Affiliations:** 1 Department of Nasopharyngeal Carcinoma, Sun Yat-sen University Cancer Center, Guangzhou, China; 2 State Key Laboratory of Oncology in South China, Collaborative Innovation Center for Cancer Medicine, Sun Yat-sen University Cancer Center, Guangzhou, China; 3 Guangdong Key Laboratory of Nasopharyngeal Carcinoma Diagnosis and Therapy, Guangzhou, China; 4 The Fifth Affiliated Hospital of Sun Yat-sen University, Guangzhou, China; 5 Translational Medicine Center, Maternal and Child Health Research Institute, Guangdong Women and Children Hospital, 521 Xing Nan Road, Guangzhou, 511400 China

**Keywords:** Nasopharyngeal carcinoma, Non-invasive diagnostic method, Methylation-specific PCR

## Abstract

**Background:**

Efforts have been made to improve the performance of nasopharyngeal carcinoma screening strategies, which include EBV related biomarkers. However, the advances achieved still remain imperfection. DNA methylation occurs early in cancer development and can be used as potential diagnostic biomarker. This study aimed to investigate the diagnostic performance of three methylated genes in nasopharyngeal carcinoma (NPC) patients.

**Methods:**

A total of 255 nasopharyngeal swabs and 35 plasma samples were collected from patients with newly diagnosed or treated NPC and healthy controls. Methylation-specific PCR (MSP) was used to evaluate the methylation levels of three genes (*SEPTIN9*, *RASSF1A*, and *H4C6*) in swabs and plasma samples. The methylation rates, sensitivity, and specificity of the candidate genes were calculated. Furthermore, the detectability of methylated genes in paired swabs and plasma was compared.

**Results:**

The detection rate of methylated *SEPTIN9*,* RASSF1A*, and *H4C6* in nasopharyngeal swabs of patients with newly diagnosed NPC was 88.2%, 92.9% and 71.8%, respectively, while it reduced to 54.3%, 42.9% and 45.7% in blood plasma. The sensitivity of detecting methylated *SEPTIN9*, *RASSF1A*, and *H4C6* to distinguish between untreated NPC and healthy controls was 88%, 93%, and 72%, respectively. Methylated *RASSF1A* showed the highest classification accuracy (AUC = 0.956). The detection rate of the methylated target genes was considerably lower in paired plasma samples.

**Conclusion:**

The detection of *RASSF1A* methylation through non-invasive nasopharyngeal cavity swab sampling demonstrates significant potential for NPC diagnosis.

**Supplementary Information:**

The online version contains supplementary material available at 10.1186/s12885-025-14508-y.

## Novelty and impact

Nasopharyngeal carcinoma (NPC) had more frequently methylated genes than normal individuals, suggesting their utility for NPC identification. There are few noninvasive tests for NPC that have been reported, and diagnostic performance is usually not very satisfactory. Nasopharyngeal swabs were collected conveniently by noninvasive means. Then we analyzed the performance of three genes methylation testing for NPC diagnosis, and the *RASSF1A* methylation assay performed best, with a sensitivity of 92.9% and specificity of 100.0%. Good findings were obtained by independently detecting *RASSF1A* methylation, which can be an independent diagnostic biomarker or even a nasopharyngeal cancer screening tool, which is inexpensive and automated.

## Posting statement

The paper is being posted on a non-commercial preprint server, including the DOI and a link to the article.

## Introduction

Nasopharyngeal carcinoma (NPC) is a malignancy originating from the nasopharyngeal epithelium. The stage of the tumor is a significant influencing factor of prognosis, with higher-stage NPC having poorer outcomes [1]. Early detection and diagnosis are particularly important in improving the cure rate and reducing recurrence of NPC.

Since infection with the highly pathogenic Epstein-Barr virus (EBV) is one of the most important etiologic factors of NPC, plasma EBV antibody and EBV DNA testing have been considered for the early detection of NPC. Two main approaches are based on enzyme linked immunosorbent assay (ELISA) EBV antibody tests and quantitative polymerase chain reaction (qPCR), detecting the antibodies responses to EBV antigens and circulating free EBV DNA. Both methods have been proven to be effective for NPC screening in high-incidence populations. However, these biomarkers have unsatisfactory positive predictive values (PPV), leading unnecessary procedures and anxiety for subjects with false positive results. Additionally, the level of circulating free DNA fragments in the blood is influenced by tumor stage and biological factors [2] which may lead to limited value of plasma EBV DNA in detecting patients with early clinical stage disease. New modalities and effective biomarkers for NPC screening is needed to reduce false positives and improve diagnostic accuracy.

DNA methylation is an epigenetic mechanism of gene silencing of tumor suppressors, which has been recognized as promising targets for the creation of robust diagnostic and predictive biomarkers. Alterations in DNA methylation usually occur in the early stages of cancer, even before changes in cell morphology and tissue structure. Abnormal DNA methylation patterns are an important feature of cancer [3, 4]. Studies have shown that methylation markers have prognostic and diagnostic significance in a variety of cancers and precancerous tissues [5, 6]. It was reported that DNA methylation is a highly stable molecular feature in NPC [7]. Compared to the plasma, nasopharyngeal swab sampling can possibly bring higher DNA concentrations, improving the sensitivity of tumor marker tests. In fact, some genes hypermethylated in NPC tissues have been reported. For example, our previous study found that *Septin9*, which is commonly used in the diagnosis of colorectal cancer, has significant difference in methylation levels between NPC tissues and benign ones [8]. In a meta-analysis, *RASSF1A* methylation is closely associated with the development of NPC [9]. *H4C6*, also known as *HIST1H4F*, is considered as a pan-oncogene and has been shown to be highly methylated in a variety of cancerous tissues including head/neck tumors but hypomethylated in benign tissues [10]. Therefore, this study investigated the clinical value of three methylated genes (*SEPTIN9*, *RASSF1A*, and *H4C6*) in nasopharyngeal swabs for NPC diagnosis.

## Materials and methods

### Study design and samples

This is a case control study with samples retrospectively collected from June 2023 till September 2023. The study flowchart is shown in Fig. 2. Three groups of subjects were screened: patients with newly diagnosed NPC (85 participants), patients with complete regression of tumor (97 participants) and healthy controls (73 participants). Patients with NPC were recruited at Sun Yat-sen University Cancer Center (SYSUCC), and the healthy controls were recruited at physical examination center. This study was approved by the Human Ethics Committee of the SYSUCC, and all participants provided informed consent.

Nasopharyngeal swabs were collected from all subjects via blind brushing method using nasopharyngeal swabs and accompanying preservation solution containing guanidine salt (Guangdong Jingquan Medical Technology Co., Ltd). In fact, this kind of swab has been successfully used for sampling during the pandemic of Covid-19. The flocked swab was inserted via the nose until the nasopharyngeal cavity was reached. Subsequently, the brush was rotated several times and quickly removed. Immediately after sampling, the brush tip was cut and placed in tubes containing 3 mL of preservation solution. Bilateral sampling was performed and swabs from the same patient were put in one tube. Furthermore, 5 mL of blood samples were collected from 35 patients and the plasma was isolated.

### DNA extraction, bisulfite conversion and methylation-specific PCR (MSP)

**Fig. 1:**
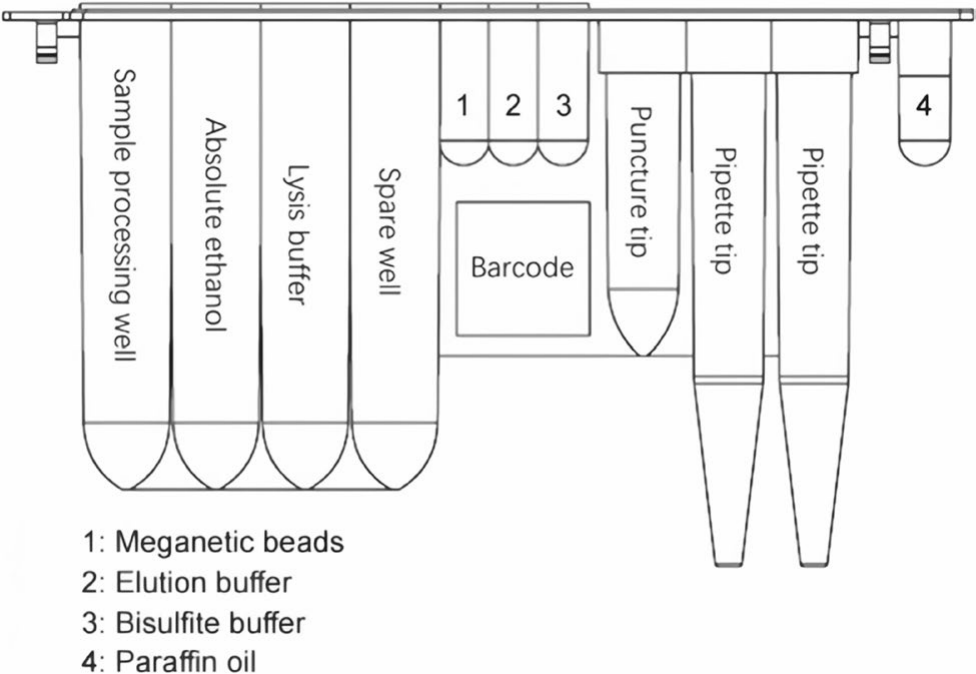
Schematic diagram of sample processing cassettes in the AutoPure-12 platform

Due to the relatively large sample size of 3 mL of preservation solution containing cells from nasopharyngeal swabs, as well as the tedious process of methylation detection, we here applied the AutoPure-12 (Guangdong Bright-Innovation BioMed Co., Ltd., Shunde, China) system to process cell preservation solution. As shown in Fig. 1, the cassettes within AutoPure-12 contain various reagents that are used for DNA extraction and bisulfite modification. This platform can maximally process 3.5 mL of liquid sample and 12 specimens at a time. The whole procedure including DNA extraction, bisulfite treatment, and purification/recovery of bisDNA (bisulfite-converted DNA) can be done automatically within 3 h, resulting in a 100 uL of bisDNA solution. Since plasma contains abundant proteins, the high viscosity leads to the aggregation of magnetic bead even use of proteinase K for protein digestion. Therefore, for plasma samples, the process of DNA isolation, bisulfite conversion and purification/recovery of bisDNA remains manually.

Methylation fluorescence quantitative kit (Guangdong Bright-Innovation BioMed Co., Ltd.) was two-channel assay consisting of each target gene along with an internal control ACTB gene. The ACTB gene served as a reference gene and samples with a cycle threshold (Ct) value of ACTB above 35 were deemed “low quality” and excluded. Ct value of methylated gene less than 40 was judged as positive. The methylation level of the target gene was determined using the difference between two Ct values (ΔCt = Ct ^ACTB^ - Ct ^target gene^), and a methylation score was calculated as 2^ΔCt^ x 100.

### Epstein–Barr virus DNA load in plasma

DNA from plasma samples was extracted using the Nucleic Acid Extraction Kit (Sansure Biotech Inc.) following the protocol recommended by the manufacturer. The EBV DNA load was measured by real-time qPCR using the EBV DNA PCR Reagents Kit (Sansure Biotech Inc.).

### Sample size calculation

This study is a diagnostic test for evaluating the accuracy of methylated genes in diagnosing NPC. Based on our preliminary experiments, the anticipated sensitivity and specificity of methylated *RASSF1A* is 80% and 90%, respectively. With an allowable error of 0.1 and a two-sided α of 0.05, the sample size is calculated with PASS software to be *N* = 70 cases. The study goal was to recruit at least 78 patients with NPC, anticipating a 10% sampling or assay failure. Actually 85 untreated NPC were recruited in this study.

### Statistical analysis

Descriptive statistical analysis was conducted for clinical and demographic characteristics. The Kruskal-Wallis test and χ2 tests were used to analyze continuous and categorical variables, respectively. The sensitivity, specificity, Youden index, positive predictive value, and negative predictive value were calculated to evaluate the performance of methylation status in NPC diagnosis. The receiver-operating-characteristic (ROC) curves were plotted based on the gene methylation score and the area under the curve (AUC) was calculated. The Mc Nemar test was used to compare the methylation status of genes in paired plasma and swab samples. Statistical analyses were performed using R Studio (publicly available). Statistical significance was defined as the conventional *P* value of < 0.05 (two-sided test).

## Results

### Patients characteristics

**Fig. 2:**
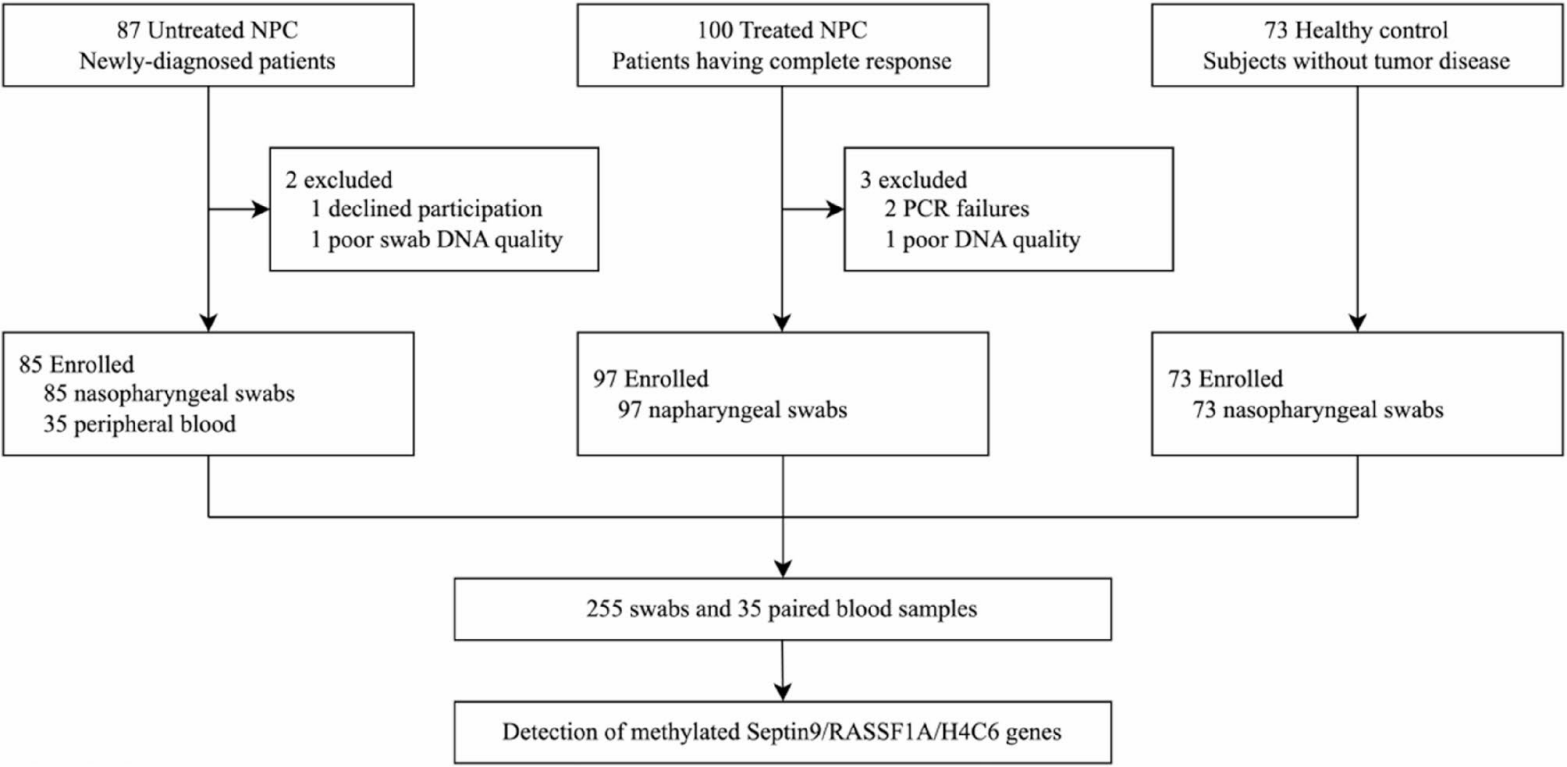
Study flow gram. Abbreviations: NPC, nasopharyngeal carcinoma; PCR, polymerase chain reaction

After excluding samples with failed quality control or PCR assays, 255 swabs were collected from 85 patients with untreated NPC, 97 patients with complete responded NPC, and 73 healthy subjects without tumor disease (Fig. 2). Paired plasma samples were obtained from 35 patients with untreated NPC. More than half of the patients had advanced stage (Stage III-IVB) (AJCC 9th) [11]. Age, gender and tumor stage were balanced between the untreated and treated group. The plasma EBV DNA was highest in patients with newly diagnosed NPC, while it was undetectable in all of the healthy controls (Table 1).

**Table 1:** Characteristics of the subjects with untreated NPC, treated NPC and healthy controls

Characteristics	Subjects			*p* value*
No.(%)/Median(Range)	Untreated NPC (*n* = 85)	Treated NPC (*n* = 97)	Healthy CTL (*n* = 73)	
Gender				0.653
Male	56 (65.9)	68 (70.1)	29 (39.7)	
Female	29 (34.1)	29 (29.9)	44 (60.3)	
Age (mean (SD))	47.29 (12.72)	46.53 (11.64)	29.29 (5.70)	0.947
T stage				0.058
T1-2	10 (11.8)	23 (23.7)	NA	
T3-4	75 (88.2)	74 (76.3)	NA	
N stage				0.922
N0-1	37 (43.5)	44 (45.4)	NA	
N2-3	48 (56.5)	53 (54.6)	NA	
M stage				1
M0	79 (92.9)	91 (93.8)	NA	
M1a-1b	6 (7.1)	6 (6.2)	NA	
Stage (AJCC 9th)				0.323
Stage IA-II	50 (58.8)	65 (67.0)	NA	
Stage III-IVB	35 (41.2)	32 (33.0)	NA	
Plasma EBV DNA (cp/ml) (median (IQR))	238.5 (1267.5)	0 (0)	0 (0)	< 0.001
Plasma EBV DNA				< 0.001
=0	23 (27.4)	88 (96.7)	21 (100)	
> 0	61 (72.6)	3 (3.3)	0 (0.0)	
SEPTIN9 methylation				< 0.001
No	10 (11.8)	55 (56.7)	45 (61.6)	
Yes	75 (88.2)	42 (43.3)	28 (38.4)	
RASSF1A methylation				< 0.001
No	6 (7.1)	94 (96.9)	73 (100.0)	
Yes	79 (92.9)	3 (3.1)	0 (0.0)	
H4C6 methylation				< 0.001
No	24 (28.2)	91 (93.8)	60 (82.2)	
Yes	61 (71.8)	6 (6.2)	13 (17.8)	
SEPTIN9 methylation score	0.39	0.00	0.00	< 0.001
RASSF1A methylation score	0.22	0.00	0.00	< 0.001
H4C6 methylation score	0.06	0.00	0.00	< 0.001

^*^The *p*-value is used to determine whether the differences between untreated NPC and treated NPC are statistically significant

### Diagnostic performance of *SEPTIN9*, *RASSF1A* and *H4C6* methylation

The detection rate and methylation scores are shown in Table 1. In swabs of newly diagnosed NPC, the highest methylated gene detection rate was 92.9% for *RASSF1A*, and the lowest for *H4C6* (71.8%). Methylated *RASSF1A* (*mRASSF1A*) was detected in 3 (3.1%) patients after treatment, and was undetectable in all of healthy controls. Sensitivity and specificity of detectable *mRASSF1A* distinguishing between untreated NPC and healthy controls were 93% and 100%, respectively (Table 2). The sensitivity of the *mSEPTIN9* and *mH4C6* was 88% and 72%, respectively, and the specificity was 62% and 82%. Besides, Table S1 shows the performance of these methylated markers distinguishing patients with untreated NPC from treated NPC patients plus healthy controls. Both of the sensitivity and specificity of the *mRASSF1A* were the highest.

**Table 2:** The performance of variables for distinguishing between untreated NPC and heathy controls

Variable	Sensitivity	Specificity	Youden index	NPV	PPV
SEPTIN9 methylation	0.88	0.62	0.50	0.82	0.73
RASSF1A methylation	0.93	1.00	0.93	0.92	1.00
H4C6 methylation	0.72	0.82	0.54	0.71	0.82
plasma EBV DNA	0.73	1.00	0.73	0.48	1.00

**Fig. 3:**
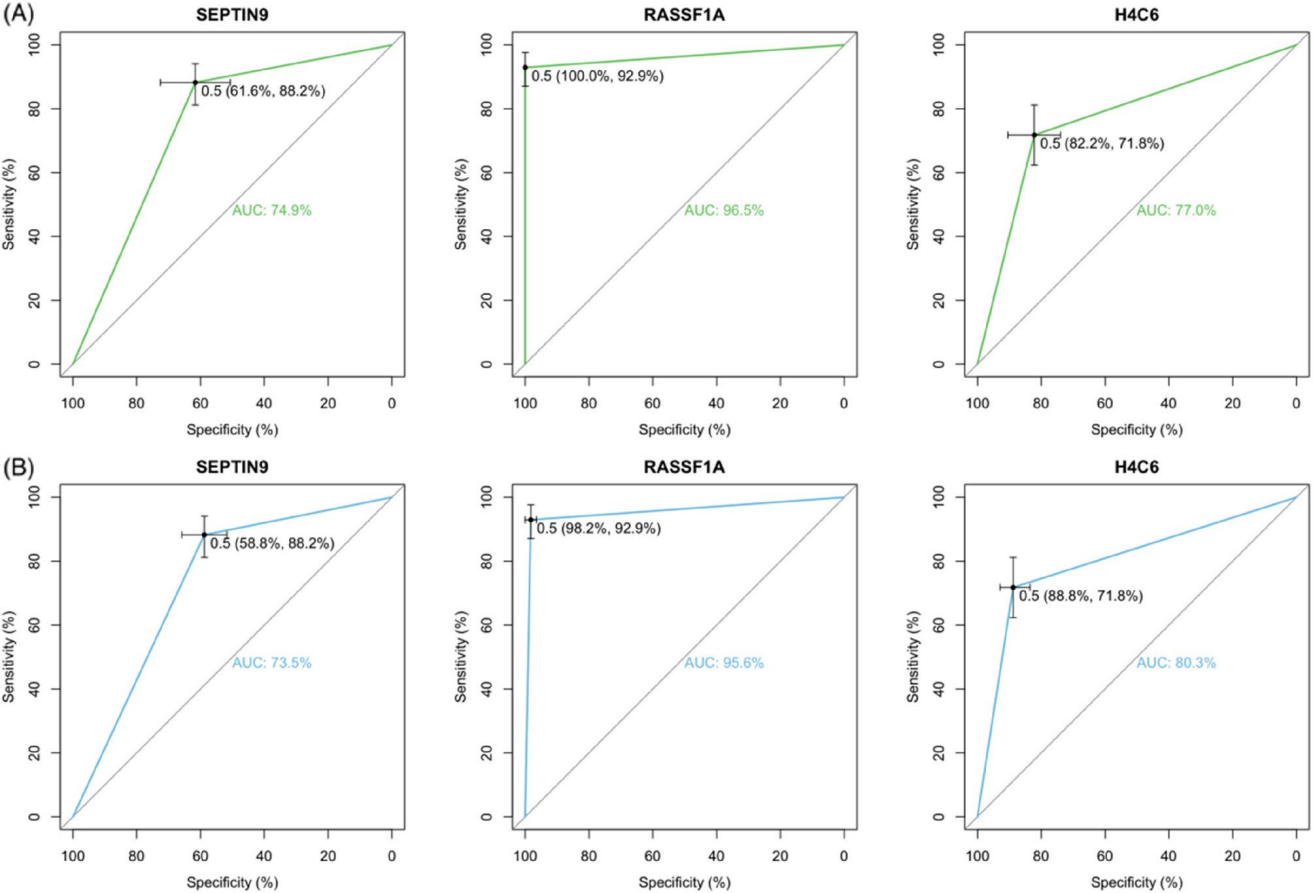
The receiver operating characteristic (ROC) curves of methylated gene detection. **A** ROC curves showing the diagnostic accuracy of methylated gene detection in differentiating between patients with NPC (*N* = 85) and healthy controls (*N* = 73). The optimal cut-off point on the ROC curve was plotted with the confidence intervals. **B** ROC curves showing the diagnostic accuracy of methylated gene detection in distinguishing between patients with NPC (*N* = 85), and patients with complete responded NPC plus healthy controls (*N* = 170). The optimal cut-off point on the ROC curve was plotted with the confidence intervals

ROC curves were constructed by the result of methylated gene detection (Fig. 3). ROC curve analysis showed AUCs of 74.9%, 96.5% and 77.0% by detecting methylated *SEPTIN9*, *RASSF1A* and *H4C6* to differentiate between untreated NPC and healthy controls. And it also showed AUCs of 73.5%, 95.6% and 80.3% to distinguish between untreated NPC, and treated NPC plus healthy controls by detecting those genes. Additionally, *RASSF1A* methylation showed the highest NPV (92%) and PPV (100%). The plasma EBV DNA was negative in 23 (27.4%) patients with untreated NPC, 88 (96.7%) patients with treated NPC and 21 (100%) healthy controls. The sensitivity and specificity of qPCR detectable plasma EBV DNA for distinguishing between untreated NPC and healthy subjects were 73% and 100%, respectively.

### Methylation difference in paired nasopharyngeal swabs and plasma

**Fig. 4:**
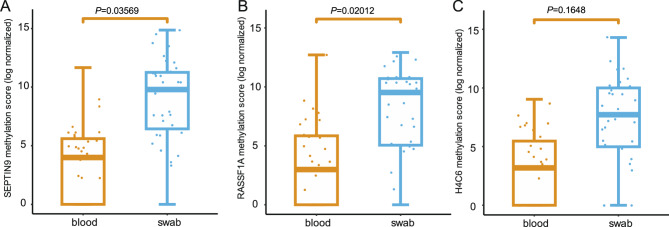
Methylation score in paired swab and blood samples. The methylation score of *SEPTIN9* (**A**) and *RASSF1A* (**B**) was significantly higher in the nasopharyngeal swab than that in the plasma sample, while no significant difference of the methylation score of *H4C6* (**C**) were found between the sample types

Paired plasma and swab samples were obtained from 35 patients. The Mc Nemar test showed significantly lower detection rate of methylated genes in the plasma samples (Table 3). Methylated *SEPTIN9*, *RASSF1A* and *H4C6* was detected in 19 (54.3%), 15 (42.9%) and 16 (45.7%) plasma samples, respectively. Moreover, the methylation score was compared between the plasma and swab samples. Significant higher scores of methylated *SEPTIN9* (*P* = 0.04) and *RASSF1A* (*P* = 0.02) were found in the swabs (Fig. 4).

**Table 3:** Identification of methylated genes in the matched swab or blood samples

Detection of methylated *SEPTIN9*					
		blood			
		negative	positive	overall	*P* value
swab	negative	0	2	2	<0.01
	positive	16	17	33	
	overall	16	19	35	

Detection of methylated *RASSF1A*					
		blood			
		negative	positive	overall	*P* value
swab	negative	3	0	3	<0.001
	positive	17	15	32	
	overall	20	15	35	

Detection of methylated *H4C6*					
		blood			
		negative	positive	overall	*P* value
swab	negative	3	3	6	<0.01
	positive	16	13	29	
	overall	19	16	35

## Discussion

Early diagnosis of NPC is quite challenging due to its anatomical peculiarity and the lack of obvious clinical signs in the early stages. At present, nasopharyngeal endoscopy combined with pathological examination of suspected cases remains the gold standard for the diagnosis of NPC, although assessment of clinical symptoms and family history provides good clues which sometimes are not specific. However, this way is mainly applicable to patients with suspected NPC and is not suitable for early screening and especially for large-scale screening due to it is costive, invasive and inconvenient. Although some reports suggest that plasma EBV DNA has high sensitivity and specificity in detecting NPC, many studies have not reached very good conclusions. For example, two studies concluded that plasma EBV DNA had a sensitivity of 84.9% and 68.8%, and a specificity of 89.7% and 88.2%, respectively, in distinguishing NPC patients from healthy individuals [12, 13]. Our study showed that plasma EBV DNA had a sensitivity of 72.6% but a specificity of 100%, which possibly due to the fact that only 21 of the healthy controls had plasma EBV DNA tested. Those false-positive results lead to repeated nasal endoscopic examination, biopsy and long-term follow-up. DNA methylation is an epigenetic mechanism of gene silencing of tumor suppressors, which has been recognized as promising targets for the creation of robust diagnostic and predictive biomarkers, more important was that the sample can be collected with noninvasive nasopharyngeal swabs.

In this study, nasopharyngeal brush sampling was performed in outpatient for newly diagnosed NPC patients (87 patients), without controlling artificially for the proportion between early NPC (Stage IA-II) and advanced NPC (Stage III-IVB). The TNM stage distribution of the patients in this study was consistent with that of initial diagnosis NPC in South China [14]. Therefore, the diagnosis performance of these newly diagnosed patients that we sampled together is reflective of the real-world situation. In this study, 115 patients with early stage NPC and 67 patients with advanced stage NPC were enrolled. We found that there was no statistical difference in methylation rate of three genes between early NPC and advanced NPC. As well, SEPTIN9 and RASSF1A methylation scores were not statistically different between early and advanced NPC. (Table S2). Further, we calculated the diagnostic performance of methylation detection rates for 3 genes for early and advanced NPC, respectively. Sensitivity of detecting *SEPTIN9*, *RASSF1A* and *H4C6* methylation in early NPC was 86%, 92% and 66%, respectively, compared to 91%, 94% and 80% in advanced NPC. Especially *RASSF1A* methylation test indicated good performance in detecting early NPC (Table S3).

In the present study, we investigated the value of methylated *SEPTIN9*, *RASSF1A* and *H4C6* in distinguishing NPC patients from normal controls. The three genes indicated a good potential to distinguish NPC from nasopharyngitis and health controls in a previous study, mainly using FFPE specimens [15]. The present study focused on real-world nasopharyngeal swab methylation detection. In the present study, the size of samples that were tested by our nasopharyngeal swabs were significantly larger, and additional analyses were performed (e.g. compared to plasma methylation tests). In the present study, we used commercial kits containing the qMS-PCR assays of the three genes. We did not quantify the nucleic acids extracted from the nasopharyngeal swabs of each participant because the process from nasopharyngeal swab DNA extraction to sulfite conversion is fully automated. However, in the final qPCR reaction, the three target genes were reacted simultaneously with the internal reference gene, ACTB gene, and the Ct value of ACTB represented the amount of DNA extracted from nasopharyngeal swabs [5, 6]. In addition, we indicated the relative content of methylated fragments of the three target genes using a methylation score formula, allowing the methylation content of the target genes in different specimens to be compared [16, 17]. We found that the methylation status and score of *RASSF1A* have high sensitivity and specificity in diagnosing NPC. Moreover, the methylation level in nasopharyngeal swab samples was higher than that in the plasma, suggesting that swab might be a better modality in NPC screening. In our pilot study, the sensitivity of bilateral nasal swab sampling was significantly higher than that of unilateral nasal swab sampling(data not shown). Because if nasopharyngeal malignancy is one-sided, and unilateral sampling will miss the contralateral tumor nidus.

Downregulated expression of *RASSF1A* has been associated with pathogenesis of cancer [18, 19]. Introducing *RASSF1A* exogenously has been shown to induce growth inhibition and apoptosis in NPC cell lines, suggesting its tumor suppressor function [20]. Previous studies with NPC tissue have implied that hypermethylation may contribute to the transcriptional inactivation of the *RASSF1A* gene [21]. In addition, hypermethylation of *RASSF1A* was correlated with advanced tumor stage and the presence of lymph node metastasis in patients with NPC [22]. Therefore, methylation tests of *RASSF1A* are of potentially valuable in improving the sensitivity for NPC detection. Our results also showed that the diagnostic sensitivity of m*RASSF1A* was over 90%, suggesting that it may aid in the screening of NPC. m*RASSF1A* was detected in 3 of 97 patients with complete responded NPC. These 3 patients in complete remission were with Stage II or III NPC, and completed treatment at least 1 year ago. As of preparing this manuscript, there were no signs of recurrence or metastasis over 15 months after the methylation tests. We will continue following these patients.

Swab sampling involves applying a brush to collect specimens of NPC in a painless and non-invasive manner, which is a relatively simple process and can be completed quickly and easily. In comparison to plasma, nasal swab can be collected without specialized training from a diverse range of populations, making it an excellent option for self-sampling in NPC screening. After the sample has been collected, it can be sent to a lab for further testing and analysis. Swabs can be used to detect the same biomarkers that were usually obtained from plasma specimens, such as EBV DNAand microRNAs [9, 23, 24]. A study in 2024 collected samples by noninvasive means such as oropharyngeal swabs, oral brushes, saliva, and gargles, and then detected their EBV DNA methylation [25]. The Youden index for detecting methylation (via the W promoter of EBV DNA) in samples collected with oropharyngeal swabs was the highest among multiple methods (sensitivity 79.7% and specificity 96.5%). Because the test required detection of EBV DNA load and EBV DNA methylation, 50.4% patients in the case group of this assay has failed to receive a diagnosis of EBV DNA methylation detection and were not included in the diagnostic performance analysis. In contrast, 97.7% of untreated NPC patients successfully underwent testing in our present study, because we detected methylation of human nasopharyngeal cells. Viral infection is only a high risk factor for cellular carcinogenesis. Medical studies have shown that EBV, HPV, and HBV are high risk factors for NPC, cervical cancer, and hepatocellular carcinoma, respectively. But, after all, cancer occurs in human cells, and these genes we tested for methylation are genes that are associated with the cancerous process in human cells [3, 4]. Therefore, if we test the methylation of human genes, it will be more indicative of cellular carcinogenesis than viruses, similar to how testing the methylation of cervical cells is closer to the reality of cellular carcinogenesis than HPV testing [26]. Methylation of nasopharyngeal cells can more directly reflect the carcinogenesis of cells compared with methylation of EBV DNA, therefore the diagnosis performance of our present study is better (sensitivity 92.9%, specificity 100.0%). Our findings indicated that the detection rate of methylated genes is significantly higher than that in the plasma for patients with NPC, probably because swab samples are close to the tumor lesion. In addition, the ingredients is relatively simple after elution of nasal swabs with preservation solution compared to plasma samples that containing abundant proteins. Thus, the nasopharyngeal epithelial cells within preservation buffer can be processed automatically for DNA extraction, bisulfite treatment, and purification/recovery of bisDNA, otherwise, manual operation would be very tedious.

## Conclusion

NPC showed more frequent methylated genes compared to normal individuals, suggesting their utility for NPC identification. *RASSF1A* methylation may be a diagnostic biomarker in swab samples that could be used for the clinical diagnosis of NPC and even for NPC screening from the perspective of cost and automation.

## Limitation

We must admit that the present study was a single-center study with a limited sample size and was retrospective, and therefore was lack of robust power to clarify the practice value in clinical situation. We conducted a multicenter prospective study starting from January 2025, including three hospitals in two provinces in China, and it was also registered on clinicaltrials.gov (NCT06367049).

## Supplementary Information

Supplementary Material 1.

Supplementary Material 2.

Supplementary Material 3.

Supplementary Material 4.

Supplementary Material 5.

Supplementary Material 6.

## Data Availability

The data of this research was submitted to RDD (Research Data Deposit) platform (http://www.researchdata.org.cn), and was currently under review. A unique RDD identification code will be provided later.

## Abbreviations

AUC: Area under the curve
EBV: Epstein-Barr virus
ELISA: Enzyme linked immunosorbent assay
m*H4C6*: Methylated *H4C6*
m*RASSF1A*: Methylated *RASSF1A*
m*SEPTIN9*: Methylated *SEPTIN9*
MSP: Methylation-specific PCR
NPC: Nasopharyngeal carcinoma
NPV: Negative predictive values
PPV: Positive predictive values
qPCR: Quantitative polymerase chain reaction
ROC: Receiver-operating-characteristic
