# Supplementary material for: Nasopharyngeal carcinoma detected noninvasively in the real world using three gene methylation analyses from automatically processed bilateral nasal swab samples
Source: BMC Cancer. 2025 Jul 5;25:1147. doi: 10.1186/s12885-025-14508-y (PMC12228209; doi:10.1186/s12885-025-14508-y)
Supplement: Supplementary file 2 — Supplementary Material 2. [file 12885_2025_14508_MOESM2_ESM.docx]

**Table S2**. The relationship between NPC stage and gene methylation.

| **Characteristics** | **Subjects** | | |
| --- | --- | --- | --- |
| **No.(%)/Median(Range)** | **Stage IA-II untreated NPC (n=50)** | **Stage III-IVB untreated NPC (n=35)** | ***p* value** |
| SEPTIN9 methylation |  |  | 0.514 |
| No | 7 | 3 |  |
| Yes | 43 | 32 |  |
| RASSF1A methylation |  |  | 1 |
| No | 4 | 2 |  |
| Yes | 46 | 33 |  |
| H4C6 methylation |  |  | 0.222 |
| No | 17 | 7 |  |
| Yes | 33 | 28 |  |
| SEPTIN9 methylation score (median (IQR)) | 0.35 (4.58) | 0.52 (3.19) | 0.883 |
| RASSF1A methylation score (median (IQR)) | 0.41 (3.91) | 0.12 (3.90) | 0.751 |
| H4C6 methylation score  (median (IQR)) | 0.02 (0.21) | 0.13 (1.12) | 0.033 |
