# Supplementary material for: Nasopharyngeal carcinoma detected noninvasively in the real world using three gene methylation analyses from automatically processed bilateral nasal swab samples
Source: BMC Cancer. 2025 Jul 5;25:1147. doi: 10.1186/s12885-025-14508-y (PMC12228209; doi:10.1186/s12885-025-14508-y)
Supplement: Supplementary file 3 — Supplementary Material 3. [file 12885_2025_14508_MOESM3_ESM.docx]

**Table S3**. The performance of variables for distinguishing between untreated NPC and heathy controls in different NPC stages.

| **Variable** | **Sensitivity** | **Specificity** | **Youden index** | **NPV** | **PPV** |
| --- | --- | --- | --- | --- | --- |
| SEPTIN9 methylation |  |  |  |  |  |
| all stages | 0.88 | 0.62 | 0.50 | 0.82 | 0.73 |
| Stage IA-II | 0.86 | 0.62 | 0.48 | 0.87 | 0.61 |
| Stage III-IVB | 0.91 | 0.62 | 0.53 | 0.94 | 0.53 |
| RASSF1A methylation |  |  |  |  |  |
| all stages | 0.93 | 1.00 | 0.93 | 0.92 | 1.00 |
| Stage IA-II | 0.92 | 1.00 | 0.92 | 0.95 | 1.00 |
| Stage III-IVB | 0.94 | 1.00 | 0.94 | 0.97 | 1.00 |
| H4C6 methylation |  |  |  |  |  |
| all stages | 0.72 | 0.82 | 0.54 | 0.71 | 0.82 |
| Stage IA-II | 0.66 | 0.82 | 0.48 | 0.78 | 0.72 |
| Stage III-IVB | 0.80 | 0.82 | 0.62 | 0.90 | 0.68 |
| plasma EBV DNA |  |  |  |  |  |
| all stages | 0.73 | 1.00 | 0.73 | 0.48 | 1.00 |
| Stage IA-II | 0.62 | 1.00 | 0.62 | 0.53 | 1.00 |
| Stage III-IVB | 0.89 | 1.00 | 0.89 | 0.84 | 1.00 |
