# Supplementary material for: Nasopharyngeal carcinoma detected noninvasively in the real world using three gene methylation analyses from automatically processed bilateral nasal swab samples
Source: BMC Cancer. 2025 Jul 5;25:1147. doi: 10.1186/s12885-025-14508-y (PMC12228209; doi:10.1186/s12885-025-14508-y)
Supplement: Supplementary file 4 — Supplementary Material 4. [file 12885_2025_14508_MOESM4_ESM.docx]

**Table S4**. The relationship between age and methylation scores of three genes.

| **Variable** | **<33y (n=87)** | **34-48y (n=87)** | **>48y (n=81)** | ***p* value** |
| --- | --- | --- | --- | --- |
| SEPTIN9 methylation score |  |  |  |  |
| untreated NPC | 0.55 | 0.20 | 1.10 | 0.46 |
| treated NPC | 0.01 | 0.00 | 0.00 | 0.66 |
| healthy control | 0.00 | 0.01 | 0.07 | 0.08 |
| RASSF1A methylation score |  |  |  |  |
| untreated NPC | 0.45 | 0.09 | 0.48 | 0.50 |
| treated NPC | 0.00 | 0.00 | 0.00 | 0.20 |
| healthy control | 0.00 | 0.00 | 0.00 | 0.75 |
| H4C6 methylation score |  |  |  |  |
| untreated NPC | 0.02 | 0.05 | 0.07 | 0.79 |
| treated NPC | 0.00 | 0.00 | 0.00 | 0.79 |
| healthy control | 0.00 | 0.00 | 0.00 | 0.28 |
