# Supplementary material for: Nasopharyngeal carcinoma detected noninvasively in the real world using three gene methylation analyses from automatically processed bilateral nasal swab samples
Source: BMC Cancer. 2025 Jul 5;25:1147. doi: 10.1186/s12885-025-14508-y (PMC12228209; doi:10.1186/s12885-025-14508-y)
Supplement: Supplementary file 5 — Supplementary Material 5. [file 12885_2025_14508_MOESM5_ESM.docx]

**Table S5**. The relationship between gender and methylation scores of three genes.

| **Variable** | **male** | **female** | ***p* value** |
| --- | --- | --- | --- |
| SEPTIN9 methylation score |  |  |  |
| untreated NPC | 0.61 | 0.21 | 0.67 |
| treated NPC | 0.00 | 0.00 | 0.45 |
| healthy control | 0.00 | 0.00 | 0.46 |
| RASSF1A methylation score |  |  |  |
| untreated NPC | 0.31 | 0.17 | 0.57 |
| treated NPC | 0.00 | 0.00 | 0.10 |
| healthy control | 0.00 | 0.00 | 0.51 |
| H4C6 methylation score |  |  |  |
| untreated NPC | 0.03 | 0.27 | 0.42 |
| treated NPC | 0.00 | 0.00 | 0.50 |
| healthy control | 0.00 | 0.00 | 0.33 |
