# Supplementary material for: Nasopharyngeal carcinoma detected noninvasively in the real world using three gene methylation analyses from automatically processed bilateral nasal swab samples
Source: BMC Cancer. 2025 Jul 5;25:1147. doi: 10.1186/s12885-025-14508-y (PMC12228209; doi:10.1186/s12885-025-14508-y)
Supplement: Supplementary file 6 — Supplementary Material 6. [file 12885_2025_14508_MOESM6_ESM.docx]

**Table S6**. The performance of variables for distinguishing between untreated NPC and treated NPC.

| **Variable** | **Sensitivity** | **Specificity** | **Youden index** | **NPV** | **PPV** |
| --- | --- | --- | --- | --- | --- |
| SEPTIN9 methylation | 0.88 | 0.57 | 0.45 | 0.85 | 0.64 |
| RASSF1A methylation | 0.93 | 0.97 | 0.90 | 0.94 | 0.96 |
| H4C6 methylation | 0.72 | 0.94 | 0.66 | 0.79 | 0.91 |
| plasma EBV DNA | 0.73 | 0.91 | 0.64 | 0.79 | 0.87 |
